# Musculoskeletal ultrasound imaging

**DOI:** 10.1186/1546-0096-12-S1-I22

**Published:** 2014-09-17

**Authors:** Paz Collado

**Affiliations:** 1 Rheumatology, Hospital Universitario Severo Ochoa, Madrid, Spain

## 

Both MSUS and MR imaging are two suitable methods used in the investigation of immature skeleton of paediatric patients. It is important to keep in mind that these **methods are complementary** instead of exclusive, acting in a synergistic way when are properly combined. MSUS is a bedside method for evaluating children **at all ages** because of **anaesthesiological support is not needed**.

From a paediatric rheumatologic perspective, the principal indication of MSUS is **direct visualization** of synovitis in peripheral joints and tendon sheaths **in few minutes**, even when clinical examination does not evidence abnormalities. But, MSUS imaging should **never replace or precede clinical evaluation**. An additional advantage of MSUS over MRI is its easy **repeatability** and capability to evaluate **a large number of joints** during a single session. The latter is of paramount importance for current ILAR classification of JIA based on the number of affected joints and the presence of particular extra-articular manifestations. Unlike MRI, MSUS is unable to display the temporomandibular joint properly.

So, far, much of the ongoing development in paediatric MSUS field has been driven by a search for solutions to clinical problems. **MSUSis usually used for a child** with articular pain, swelling, or mechanical symptoms, **without definitive diagnosis on clinical examination, in order to elucidate the diagnosis at peripheral joints**. MSUS is also used to guide needle injection. Table 1 lists the main detectable musculoskeletal diseases by ultrasound in daily practice. Currently the principal applications for using MSUS in patients with JIA include: detection of synovitis, tenosynovitis, enthesitis and cartilage and bone abnormalities. To date, the role of MSUS in therapy monitoring has not been fully established.

The challenge to assess synovitis has been minimized thank to technology for MSUS equipments has evolved considerably. Nevertheless, **real time (or dynamic) examination** is the most reliable method to distinguish quick and early between synovitis and joint cartilage leading to immediate improvement of the diagnosis and initial therapy decision of articular disorders. The MSUS appearance of tenosynovitis and enthesitis on B-mode is the same in all patients with inflammatory disease irrespective of age.

As the ossification centre could show a smooth surface or irregular surface, the presence of other pathologic features along with erosion avoids misdiagnosis. MRI has proven earlier detection of bone abnormalities than MSUS. To date, **the role of Doppler** technique **has not been entirely establishedyet**.

Future topics for study include: establishing international definitions for joint components in healthy children and for MSUS findings in JIA patients, consensus on scanning protocols and scoring systems, evaluation of the role of MSUS with power Doppler in the assessment of the real state of disease (activity /remission) and developing a specific training programme for paediatric rheumatologists performing US in patients with JIA.

## Disclosure of interest

None declared.

